# *Nigella sativa* oil protects against cadmium-induced intestinal toxicity via promotion of anti-inflammatory mechanisms, mucin expression and microbiota integrity

**DOI:** 10.22038/AJP.2021.18774

**Published:** 2022

**Authors:** Akinleye Stephen Akinrinde, Abimbola Olumide Adekanmbi, Folake Olayinka Olojo

**Affiliations:** 1 *Environmental and Gastrointestinal Toxicology Laboratory, Department of Veterinary Physiology and Biochemistry, Faculty of Veterinary Medicine, University of Ibadan, Oyo State, Nigeria*; 2 *Environmental Microbiology and Biotechnology Laboratory, Department of Microbiology, Faculty of Science, University of Ibadan, Oyo State, Nigeria*; 3 *Department of Chemistry, Faculty of Science, The Polytechnic, Ibadan, Oyo State, Nigeria*

**Keywords:** Nigella sativa, Cadmium chloride, MUC2, TNFα, IL-2, Number of goblet cells Microbiome

## Abstract

**Objective:**

This study examined the protective effects of *Nigella sativa* oil (NSO) on cadmium (Cd)-induced alterations affecting gut morphology and microbiota composition, as well as the involvement of mucus glycoprotein (MUC2) and immuno-inflammatory markers (TNFα and IL-2) in the colon of rats.

**Materials and Methods:**

Male Wistar rats, randomized into four groups, were treated either with distilled water (control), CdCl_2 _(100 mg/kg), CdCl_2_+NSO (1 ml/kg) or NSO alone. After the experiments, faecal samples were processed for microbial culture on various selective media, while intestinal segments were prepared for histopathological examination and immunohistochemistry. The composition of NSO was analyzed using Gas Chromatography-Mass Spectrometry (GC-MS).

**Results:**

Oral Cd administration provoked dramatic increases in faecal counts of potentially pathogenic bacteria (Staphylococci, Enterococci, *Pseudomonas* and *Escherichia coli*)*, *while decreasing probiotic lactobacilli counts. Cadmium treatment caused down-regulation of colonic MUC2 (p=0.003) and IL-2 (p=0.03), but increased TNFα (p=0.034), along with reduced goblet cell counts and mucus production. Conversely, treatment with NSO significantly improved Lactobacilli counts (p=0.042), while reducing the levels of potentially pathogenic species. In addition, NSO significantly restored colonic expressions of MUC2 (p=0.001), TNFα (p=0.007) and IL-2 (p=0.025) to control levels. GC-MS analysis of NSO revealed the presence of the active ingredient, thymoquinone and a high content of unsaturated fatty acids, including trans-13-octadecenoic acid and oleic acid.

**Conclusion:**

This study highlights the intestinal mucus, microbiota and immuno-inflammatory system as important protective targets of NSO against Cd-induced intestinal toxicity.

## Introduction

Exposure to environmental stressors such as cadmium (Cd) may lead to an impairment of the structural and/or molecular components of the intestinal barrier, which essentially includes, at least, mucus layer, a single layer of epithelial cell barrier with associated tight and adherens junctions, a sub-epithelial connective tissue layer and the homeostasis of commensal bacteria and other potentially pathogenic species (Mosca et al., 2016 ▶; Nagpal and Yadav, 2017 ▶). Colonic mucus, composed of water, glycoproteins (mucin), electrolytes, lipids and other components, is a dynamic biophysical barrier that provides lubrication and protection for the underlying epithelium and consists of two layers, an adherent inner layer and a loosely-bound outer layer (Varum et al., 2011 ▶). While commensal bacteria normally inhabit the outer mucus layer, utilizing mucins as energy source, they are not normally found in the adherent mucus layer (Li et al., 2015 ▶). 

Commensals in the gut lumen are mainly anaerobic resident microbes (e.g. Lactobacilli and Bifidobacteria), which normally provide resistance against invasion and/or colonization by potentially pathogenic species (e.g. Enterobacteriaceae, *Pseudomonas*, *E. coli*, etc.) and thereby protect the host (van Vilet et al., 2010 ▶). However, the delicate balance of the gut microflora may be disrupted during exposure to toxicants including heavy metals such as Cd, resulting in overgrowth of pathogenic species on epithelial cells (Liu et al., 2014 ▶). These alterations in bacterial activity and populations (dysbiosis) under stressor-induced conditions may result in increased degradation of the outer mucus layer, leading to the development of intestinal inflammation and penetration of commensal and potentially pathogenic bacteria into the inner mucus layer (Larsson et al., 2011 ▶). 

Although much attention has focused on the bioaccumulation and toxicity of Cd in organs such as the liver and kidney, it is now known that the gut is also an important target for Cd toxicity (Tinkov et al., 2018 ▶). Indeed, studies have shown, for example, that only a very small fraction (about 0.5-3.0%) of dietary Cd is actually absorbed in animal species (Asagba, 2013 ▶), suggesting that a much larger portion of ingested Cd could be available for interaction with gut components and microbes, especially in the most distal segments such as the colon. Existing data has shown that Cd induces significant gut dysbiosis with increased lipopolysaccharide production. This has also been associated with increased inflammatory responses and increased gut permeability and possible bacterial translocation (Liu et al., 2014 ▶). However, very little is known about the effects of Cd exposure on mucus production and mucin synthesis in the colon. Studies have shown that alterations in the integrity of the mucus layer and the homeostasis of gut microbial composition and physiology are associated with several negative health effects, including susceptibility to infections and inflammatory bowel diseases (Cameron and Sperandio, 2015 ▶). Therefore, knowledge of the impact of Cd exposure on critical components of the mucosal barrier may help to develop therapeutic and preventive strategies to manage gut abnormalities.

The essential oil derived from Black cumin seed (*Nigella sativa*), used as edible oil in parts of Africa and the Middle East, has been reported to possess several beneficial pharmacological properties, including antioxidant, anti-inflammatory, antimicrobial, immune-enhancing and anticancer effects (Alenzi et al., 2010 ▶). The biological activities shown by the seed oil have been largely attributed to the presence of the bioactive agent, thymoquinone, which has been shown to possess radical scavenging activity and an ability to inhibit several mediators of inflammation (Khan and Afzal, 2016 ▶). 

In this study, we sought to understand the effects of Cd exposure on the composition of gut microbiota, as well as qualitative and quantitative alterations that occur in the mucous layer, mucin (MUC2) production and gut inflammation in rats. In addition, we sought to investigate the protective effects of *N. sativa* oil on Cd-induced alterations in mucosal integrity, gut inflammatory status and microbiota homeostasis in the rats.

## Materials and Methods

**Chemicals**

Cadmium chloride (CdCl_2_) was purchased from Sigma Chemical Co. (St. Louis, MO, USA). Black seed (*N. sativa*) oil was obtained commercially as a product from Hemani herbal LLC (Longwood, FL, USA).

**Animals**

Twenty-eight (28) male Wistar albino rats weighing between 100-130 g were obtained from the Experimental Animal Unit of the Faculty of Veterinary Medicine, University of Ibadan, Nigeria. They were housed in plastic cages in a well-ventilated animal house facility at the Department of Veterinary Physiology and Biochemistry, University of Ibadan, where they had unhindered access to a commercial rat feed and distilled water, free of Cd or other metal contaminants. The rats were allowed to acclimatize to the animal house conditions for a week before commencement of the experiments. The rats were subjected to a natural photoperiod of 12 hr light: dark cycle throughout the experimental period. All the rats were handled humanely according to the guidelines in the ‘Guide for the Care and Use of Laboratory Animals’ document prepared by the National Academy of Science and published by the National Institute of Health (PHS, 1996 ▶). The protocol followed was approved by the Animal Care and Use Research Ethics Committee (ACUREC) of the University of Ibadan.

**Experimental and Treatment protocols**

The rats were randomly assigned to four groups of seven rats per group as follows:Group A: Control rats that received distilled water alone orally at 3 ml/kg for 7 consecutive days. Group B (Cd only): Rats treated with CdCl_2_ at 100 mg/kg by oral gavage for 7 consecutive days. Group C (Cd+NSO): Rats treated with CdCl_2 _at 100 mg/kg by oral gavage and concurrently with *Nigella sativa* oil (NSO) orally at 1 ml/kg for 7 consecutive days. Group D (NSO only): Rats treated with *Nigella sativa* oil alone at 1 ml/kg for 7 consecutive days.

The doses of CdCl_2_ (Liu et al., 2014 ▶) and NSO (Alenzi et al., 2010 ▶) were chosen based on previous studies.

At the end of the experiment, freshly-voided faeces from each rat were collected aseptically into sterilized plain sample bottles containing 10% glycerol and then processed for microbiota analysis within 30 min after collection. Thereafter, the body weight of the rats was measured prior to their euthanasia by cervical dislocation and the small intestine and colon were quickly isolated and flushed with cold physiological saline. The duodenum and ileum were separated as the proximal 10 cm and the distal one-third of the small intestine, respectively.

**Faecal bacteriologic analysis**

Faecal samples were weighed and homogenized in tryptone soy broth and serially diluted in 10-fold for bacterial counts. Aliquots (1000 µl) of the selected dilution factors of each sample were analyzed quantitatively using the standard pour plating technique (Harrigan and MacCance, 1996 ▶) for groups of bacteria, including Lactobacilli, Staphylococci, Pseudomonas, Enterococci and Coliforms. The set-up was incubated at 35±2ºC for 24-48 hr under aerobic conditions, with the exception of De Man Rogosa and Sharpe plates, which were incubated under anaerobic conditions. The different culture media used for the selective isolation of different groups of bacteria were: MacConkey agar (Oxoid, Basingstoke, United Kingdom) for coliforms; Eosin Methylene Blue agar (Oxoid, Basingstoke, United Kingdom) for *Escherichia coli*; Slanetz and Bartley medium (Oxoid, Basingstoke, United Kingdom) for Enterococcus; Centrimide agar (Merck, Germany) for *Pseudomonas aeruginosa; *Mannitol Salt agar (Oxoid, Basingstoke, United Kingdom) for Staphylococcus, De Man Rogosa and Sharpe agar (Himedia, Mumbai, India) for Lactobacillus. Bacterial count is expressed as colony-forming units per gram (CFU/g) faeces and was recorded against each sample.

**Histopathology of duodenum, ileum and colon**

Specimens of the duodenum, ileum and colon were fixed in 10% buffered formalin and processed for paraffin sections of about 5-6 µm thickness. The sections were mounted on glass slides and then stained with Haematoxylin and eosin (H&E) and Periodic acid Schiff (PAS) stains (Drury and Wallington, 1976 ▶; Avwioro, 2010 ▶). The slides were thereafter examined using light microscopy.

**Immuno-histochemical analysis of colonic MUC2, TNFα and IL-2**

Immunohistochemical studies were carried out according to methods described by Todorich et al. (2011) ▶ using formalin-fixed, paraffin-embedded colonic sections mounted on positively charged slides using avidin-biotin-peroxidase immune-histochemical staining for MUC2, TNFα and IL-2, according to the manufacturer’s instructions on the kits. The slides were initially deparaffinized in xylene and then re-hydrated with graded alcohol concentrations (100%-75%). Antigen retrieval was performed by immersing the slides in citrate buffer and incubating them at 95-100ºC for 25 min. After cooling the slides, peroxidase quenching was performed using 3% H_2_O_2 _in methanol, v/v. Thereafter, non-specific antigen binding was blocked by adding 10% fetal bovine serum in phosphate buffered saline (PBS) onto the slides. This was followed by addition of appropriately diluted primary antibodies to the sections on the slides and then incubation in a humidified chamber at room temperature for 1 hr. Detection of bound antibody was carried out using appropriately diluted Antibody amplifier+Polymer- HRP Micro-Polymeric-HRP secondary antibody, followed by addition of diaminobenzidine (DAB) substrate solution (freshly made just before use: 0.05% DAB - 0.015% H_2_O_2_ in PBS) to reveal the color of antibody staining. The slides were thereafter, counterstained in haematoxylin, and were subsequently dehydrated through 4 times of incubation in alcohol (95%, 95%, 100% and 100%) for 5 min each. The slides were cleared in xylene and coverslips were applied using mounting solution. The immune-reactive positive expressions of MUC2, TNFα and IL-2 antibody staining were viewed starting from low magnification on each slide then with 400× magnifications, using a light microscope (Olympus) and a digital camera.

**Gas chromatography-mass spectrometry (GC-MS) analysis of black seed oil**

The chemical analysis of the components of black seed oil was performed by gas chromatography-mass spectrometry, using an Agilent 6890 gas chromatography system coupled with an Agilent 5975 mass selective detector (Chemetrix, Pty, Ltd, Agilent Technologies, DE, Germany), according to methods described by Akinrinde et al (2019) ▶, with slight modifications. The GC column was a Zebron -5MS column (consisting of cross-linked 5% phenylmethylpolysiloxane; ZB-5MS 30 m × 0.25 mm × 0.25 μm). The carrier gas was GC grade helium maintained at a flow rate of 2 ml/min; volume injections were 1 ml splitless injections with injector temperature and source temperature set at 280ºC. Column temperature ranged between 70 and 270ºC, ramped at intervals 15 or 20°C/min. Data were gathered with ChemStation Integrator and the identification of oil components was based on computer matching with mass spectra stored in the NIST11.L library. The relative percentage of oil constituents was calculated from the peak areas.

**Statistical analysis**

Data are expressed as mean±standard deviation and analyzed using GraphPad Prism software (Version 7.00). The differences among means were assessed using One way Analysis of Variance (ANOVA) followed by the Tukey’s *post hoc* test for multiple comparisons across the groups. p values <0.05 were considered statistically significant. The mean area% of MUC-2, TNFα and IL-2 was quantified in 10 images from 5 rats in each group using Image J (version 1.46r; National Institutes of Health, USA). 

## Results

**Chemical composition of** ***N. sativa*** **seed oil**

**Figure 1:**
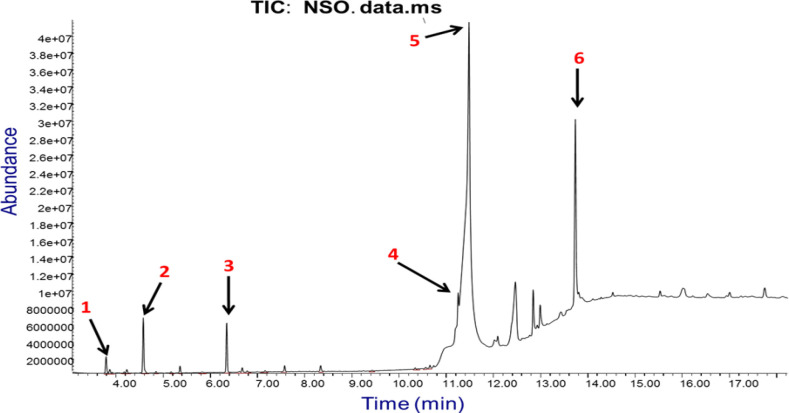
GC-MS chromatogram of *Nigella sativa* seed oil. Major peaks identified are: (1) β-thujene (RT=3.802 min); (2) o-Cymene (RT=4.590); (3) Thymoquinone (RT=6.357); (4) Oleic acid (RT=11.260); (5) Trans-13-octadecenoic acid (RT=11.488) and (6) E, Z-1, 3, 12, nonadecatriene-5,14-diol (RT=13.741)

The chemical constituents of the commercially obtained black seed oil preparation used in the present study, with their retention indices are presented in Table 1, while the total ion chromatogram showing the major peaks is depicted in Figure 1. A total of 12 compounds, representing 89.48% of the total oil composition, were identified by GC-MS. It was observed that the most abundant compounds were trans-13-octadecenoic acid (57.81%), E, Z-1, 3, 12-nonadecatriene-5, 14-diol (15.04%) and oleic acid (11.62%), in respective order. Other notable compounds identified were o-cymene (1.87%), and the usual active component of *N. sativa*, thymoquinone (1.60%). The remaining components were terpenoids, including β-thujene, α-pinene, γ-terpinene, carvacrol and longifolene, which were detected at relatively low amounts. 

**Effect of** ***N. sativa*** **oil (NSO) on body weights in Cd-treated rats**

**Figure 2:**
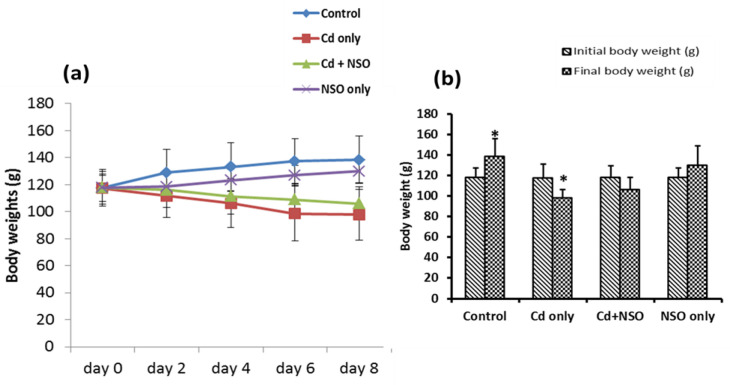
Effects of *Nigella sativa* oil on body weight of cadmium-treated rats. Values are expressed as mean±standard deviation (n=7); *p<0.05 when compared with the average initial weight

The effects of NSO on the body weights of Cd-treated rats are presented in Figure 2. Oral administration of Cd led to a progressive decline in the body weights of rats over the course of the experiment, compared to the control rats, which exhibited progressive gain in body weight (Figure 2a). Specifically, rats treated with Cd alone showed a significant (p<0.05) decline in the body weights by 19.94%, in contrast to the control rats, which exhibited a significant (p<0.05) increase in the body weights by 15.03% during the experimental period (Figure 2b).

**Table 1:** Chemical constituents of *Nigella sativa* seed oil

S/N	Compounds identified	Chemical class	Molecular formula	Area (%)	RT
**1**	β-thujene	Monoterpene	C_10_H_16_	0.59	3.802
**2**	α-pinene	Monoterpene	C_10_H_16_	0.16	3.881
**3**	β-pinene	Monoterpene	C_10_H_16_	0.14	4.241
**4**	o-Cymene	Aromatic hydrocarbon	C_10_H_14_	1.87	4.590
**5**	γ-terpinene	Monoterpene	C_10_H_16_	0.05	4.860
**6**	Trans-4-methoxy thujane	Terpenoid	C_8_H_16_O_2_	0.23	5.368
**7**	Thymoquinone	Benzoquinone	C_10_H_12_O_2_	1.60	6.357
**8**	Carvacrol	Monoterpenoid	C_10_H_14_O	0.16	6.686
**9**	Longifolene	Sesquiterpene	C_15_H_24_	0.21	7.584
**10**	Oleic Acid	Monounsaturated Omega-9 fatty acid	C_18_H_34_O_2_	11.62	11.260
**11**	Trans-13-Octadecenoic Acid	Monounsaturated fatty acid	C_18_H_34_O_2_	57.81	11.488
**12**	E,Z-1,3,12-Nonadecatriene-5,14-diol	Polyunsaturated fatty acid	C_19_H_34_O_2_	15.04	13.741
**Total identified**				89.48%	

RT=Retention time

Although rats co-treated with Cd and NSO also showed progressive weight loss up to 11.15% during the experimental period, the difference between the average initial and final weights was not statistically significant. Similar to the control rats, rats treated with NSO alone showed a progressive increase in body weight amounting to a percentage increase of 9.35%, although the difference between the final and initial body weights of this group was not statistically significant. 

**Effect of** ***N. sativa*** **oil (NSO) on changes in intestinal microflora in Cd-treated rats**

**Figure 3:**
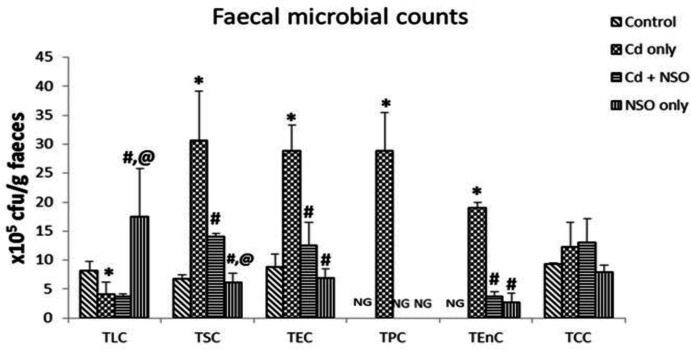
Effects of *Nigella sativa* oil on intestinal microbiota composition in cadmium-treated rats. Data are presented as mean±standard deviation (n=5). ^*^p=0.041, 0.02, 0.033, 0.008 and 0.0003 for TLC, TSC, TEC, TPC and TEnC, respectively, compared to the control group; ^#^p=0.042, 0.039, 0.032, 0.008 and 0.0006, respectively for TLC, TSC, TEC, TPC and TEnC compared to the Cd only group; ^@^p=0.005 and 0.004 for TLC and TSC, respectively, compared to the Cd+NSO group. TLC: Total lactobacilli count; TSC: Total Staphylococcal count; TEC: Total *E. coli* count; TPC: Total Pseudomonas count; TEnC: Total enterococcal count; TCC: Total coliform count; and NG, no growth

As depicted in Figure 3, rats treated with Cd alone had significantly (p<0.05) higher counts of Staphylococci, *E. coli*, *Pseudomonas* and enterococci, when compared with the control group. In contrast, Lactobacilli count was significantly (p = 0.041) reduced in the Cd-treated group compared to the control group. 

However, in rats co-treated with NSO, there were significantly (p<0.05) lower counts of Staphylococci (p = 0.039), *E. coli *(p = 0.032) and enterococci (p = 0.0006) as compared to the group treated with Cd alone, while Lactobacilli count significantly (p = 0.042) increased. Notably, *Pseudomonas* and enterococci were not detected in faecal samples of the control rats, and *Pseudomonas* was also not detected in faecal samples from rats treated with NSO alone. Overall, Cd exposure resulted in lower counts of Lactobacilli and higher counts of Staphylococci, *E. coli*, *Pseudomonas* and enterococci, while NSO treatment reversed these effects.

**Morphology of duodenum, ileum and colon**

**Figure 4:**
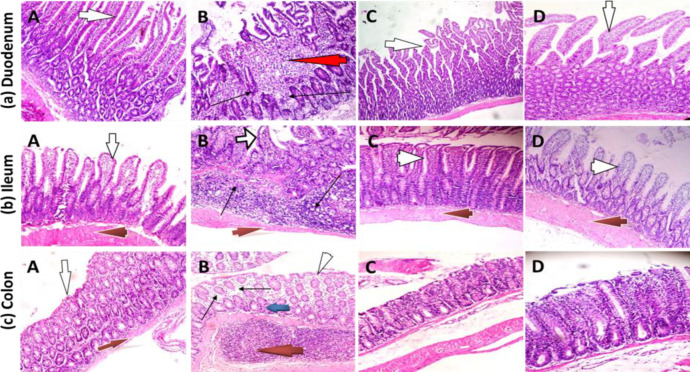
Photomicrographs of the duodenum, ileum and colon in rats treated with cadmium chloride and *Nigella sativa* oil (H&E). A, control group; B, Cd only group; C, Cd+NSO group; D, NSO only group. (a) Duodenum: Villi (white arrows) morphology was largely normal in control, Cd+NSO and NSO groups; focal areas of ulceration (red arrow) and inflammatory cell infiltration (black arrows). (b) Ileum: Normal villi (white arrows) in all the groups, except the Cd group, which showed distorted villi arrangement and inflammatory cell infiltration (black arrow) in the sub-mucosal layer. Muscularis layer (red arrows) appears normal in all the groups (c) Colon: epithelium appears largely normal in the control, Cd+NSO and NSO groups. Lymphocytic infiltration (black arrows) and several lymphoid aggregates (red arrow) in the sub- mucosal layer

The photomicrographs of histological sections of the duodenum, ileum and colon in the experimental rats are presented in Figure 4. Sections from the duodenum, ileum and colon of the control rats (Plate marked A) revealed well preserved mucosal integrity with no evidence of villi distortion or cellular degeneration, inflammation or necrosis. In rats treated with Cd only (Plates marked B), the duodenum showed mucosal layer with focal areas of ulceration, distortion of villi architecture and severe infiltration of the lamina propria and sub-mucosal layer by inflammatory cells. The ileum sections in this group of animals also showed mucosal layer with inflamed villi with severe inflammatory cell infiltration of the lamina propria and sub-mucosal layers. The sub-mucosal glands showed moderate degeneration with appearance of typical cells. In the same vein, the colonic mucosa of the Cd-treated rats showed moderate infiltration of inflammatory cells made up predominantly of lymphocytes and a few polymorphs. In rats concurrently treated with Cd and NSO (Plates marked C), the duodenal mucosa and villi were moderately preserved with only mild cellular infiltration of the lamina propria, while mucosal glands appeared normal.

Similarly, the ileal sections in this group of rats showed moderately preserved mucosa with mild inflammatory cell infiltration. The colonic mucosa was well preserved in this group of rats with only mild cellular infiltration in the lamina propria. Treatment of rats with NSO only (Plates marked D) did not produce noticeable deleterious effects as the mucosal layers of the duodenum, ileum and colon appeared largely normal with normal villi and sub-mucosal layers. In all the groups, the muscularis layer appeared unaffected by the different treatments.

**Effect of** ***N. sativa*** **oil (NSO) on colonic mucus content in Cd-treated rats**

**Figure 5:**
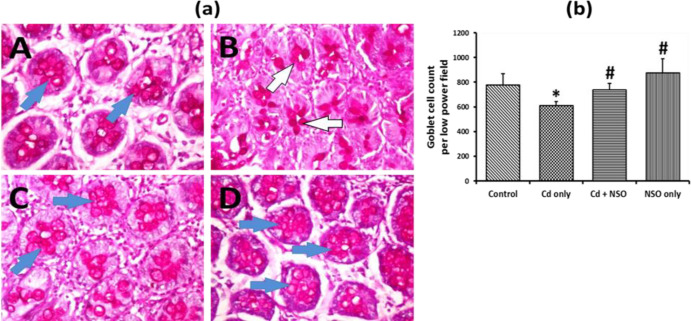
(a) Photomicrograph of colonic sections stained by Periodic Acid Schiff technique. Blue arrows indicate areas of high intracellular mucin production; white arrows indicate areas of low intracellular mucin production. A, control; B, Cd only; C, Cd+NSO; D, NSO only. (b) Quantification of goblet cell numbers in low power field of colonic sections of rats treated with cadmium and *Nigella sativa* oil. *indicates significant difference (p=0.0064) compared to the control group; ^#^indicates significant differences (p=0.036 and 0.015 for the Cd+NSO and the NSO group) compared to the Cd only group

Periodic Acid Schiff (PAS) histology staining of the colonic mucosa of the experimental rats is presented in Figure 5. The colons of control rats showed normal mucus production with abundant goblet cells (Figure 5a; Plate A). In contrast, there was a significant (p = 0.0064) reduction in PAS positivity (Figure 5a; Plate B) and goblet cell numbers (Figure 5b) in rats treated with Cd alone, when compared with the control rats. However, in contrast to rats treated with Cd alone, co-treatment with NSO led to augmented mucus production and a significant (p = 0.036) increase in goblet cell numbers. Notably, rats treated with NSO alone showed higher degrees of colonic PAS staining compared to rats treated with Cd alone, and significantly (p<0.05) higher goblet cell numbers above the control levels.

**Immuno-histochemical staining of colonic MUC2**

**Figure 6:**
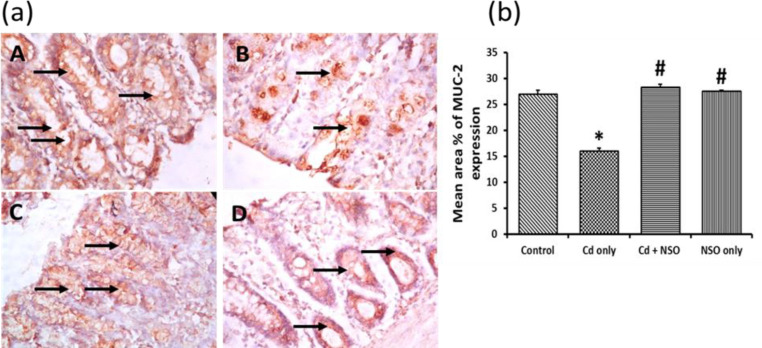
(a) Immuno-histochemical staining of MUC 2 in colonic sections of rats treated with cadmium and *Nigella sativa *oil X400; MUC 2 expression is indicated as golden brown coloration (black arrows) on tissue structures. A, control; B, Cd only; C, Cd+NSO; D, NSO only. (b) The mean area% of MUC 2 expression in the different groups. *indicates significant difference (p=0.003) compared to the control group; ^#^indicates significant differences (p=0.010 for Cd+NSO group and 0.008 for NSO group) compared to the Cd only group

Immunohistochemical evaluation of distal colon segments from untreated rats showed that MUC2 was constitutively expressed (indicated by dense brown staining in the tissues) in the crypts up to the surface epithelium, whereas during Cd treatment, MUC2 level was substantially reduced (Figure 6). The mean area% of MUC2 expression in the control rats was significantly (p = 0.003) higher than that of the Cd-treated rats. Treatment with NSO alone or in combination with Cd significantly improved MUC2 expression compared to the rats treated with Cd alone.

**Immuno-histochemical staining of colonic tumor necrosis factor alpha (TNFα)**

**Figure 7:**
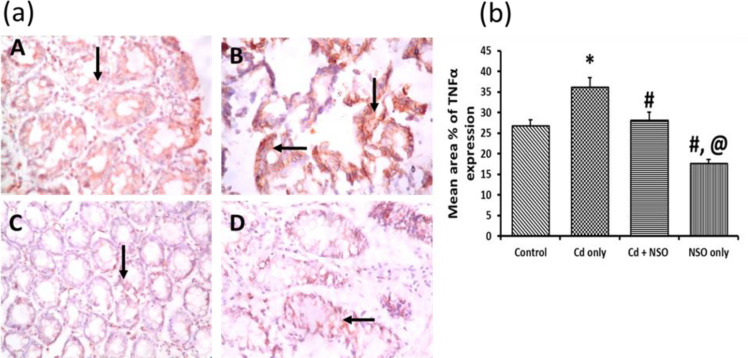
(a) Immuno-histochemical staining of Tumor Necrosis Factor (TNFα) in colonic sections of rats treated with cadmium and *Nigella sativa *oil X400; TNFα expression is indicated as golden brown coloration (black arrows) on tissue structures. A, control; B, Cd only; C, Cd+NSO; D, NSO only. (b) The mean area% of TNFα expression in the different groups. *indicates significant difference (p=0.029) compared to the control group; ^#^indicates significant differences (p=0.035 for Cd+NSO group and 0.017 for the NSO group), compared to the Cd only group; ^@^indicates significant difference (p=0.023) compared to the Cd+NSO group

Immunohistochemical staining of TNFα in the colon segments is depicted as brown reactions in the cytoplasm and connective tissues as shown in Figure 7. The control rats showed relatively lower colonic TNFα expression, compared to the Cd-treated rats, which showed intense TNFα staining and significantly higher (p = 0.029) mean area% of expression compared to all the other groups. On the contrary, the mean area% of TNFα expression was significantly (p = 0.035) decreased in the rats treated concurrently with NSO, and more so in the rats administered with NSO alone.

**Immuno-histochemical staining of colonic interleukin-2 (IL-2)**

**Figure 8:**
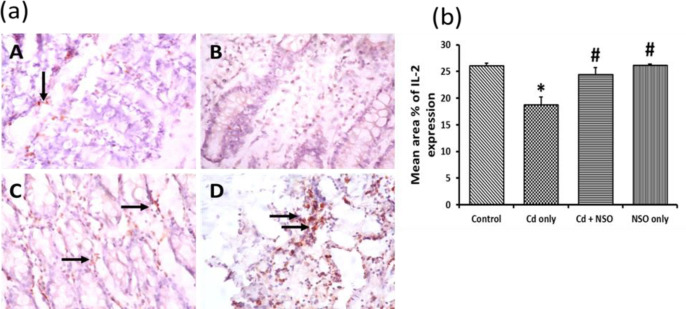
(a) Immuno-histochemical staining of interleukin-2 (IL-2) in colonic sections of rats treated with cadmium and *Nigella sativa *oil X400; IL-2 expression is indicated as golden brown coloration (black arrows) on tissue structures. A, control; B, Cd only; C, Cd+NSO; D, NSO only. (b) The mean area% of IL-2 expression in the different groups. *indicates significant differences (p=0.032) compared to the control group; ^#^indicates significant differences (p=0.025 and 0.041 for the Cd+NSO and NSO groups, respectively) compared to the Cd only group

The photomicrographs and mean area% of IL-2-positive staining for all groups are presented in Figure 8. There was a significant (p = 0.032) reduction in colonic IL-2 expression in the Cd-treated group, compared to the control rats. However, with NSO treatment, there was restoration of IL-2 levels to the control levels as indicated by increased IL-2 staining and increased mean area% of IL-2 expression.

## Discussion

Cadmium has a long history of recognition as an environmental pollutant with serious toxic effects on various body tissues. However, the impact of Cd exposure on gastrointestinal structures has gained attention more recently, as the heavy metal is now known to induce serious disturbances that affect gut physiology, epithelial integrity, and gut microbiota, as well as mucus production (Liu et al., 2014 ▶; Tinkov et al., 2018 ▶). In the present study, we focused on investigating the protective effects of *N. sativa* oil on different aspects of intestinal physiology such as mucus production, mucin synthesis, inflammatory status and microbiota composition in rats. We found that oral administration of Cd at 100 mg/kg increased faecal counts of potentially pathogenic bacteria, such as staphylococci, enterococci, *Pseudomonas* and *E. coli, *while reducing lactobacilli count. These microbial changes were associated with reductions in colonic mucus production, reduced MUC-2 and IL-2 expression but increased TNFα. On the other hand, administration of NSO at the dose of 1 ml/kg daily significantly promoted intestinal protection against the Cd-induced changes.

The present data indicated significant reductions in body weight gain in the Cd-treated rats, which was more pronounced in those treated with Cd alone, but alleviated in rats treated concurrently with NSO. This observation of acute Cd-induced body weight loss is in agreement with an earlier report indicating that Cd promotes an early stress response correlating with increased oxidative stress, secretion of serum cortisol and increased catabolism of tissue proteins within the first week of exposure (Osifo and Iyawe, 2018 ▶). More relevant to the present study was our observation of reduced appetite, reduced feed intake and consequent decreases in body mass of Cd-treated rats. This may be related to some neurological disturbances modifying the functioning of the appetite-regulatory system, including the hypothalamic-pituitary-adrenocortical axis, as previously reported (Perry et al., 2019 ▶). Reduced weight gain in chemical-induced toxicity has also been attributed to impairment of nutrient digestion, absorption and utilization resulting from damage to the intestinal epithelium (Chauhan et al., 2011 ▶). As further obtained in this study, intestinal epithelial damage observed during histopathological examination of the duodenum and ileum in Cd-exposed rats provided evidence that an impairment of digestion and absorption of nutrients could have indeed contributed to the observed body weight losses.

Results from several studies have indicated that the gut microbiota must be considered a crucial target of the toxic effects of many heavy metals, including Cd (Liu et al., 2014 ▶; Jin et al., 2017 ▶). There is evidence that probiotic bacteria such as Lactobacilli and Bifidobacteria, which form the predominant anaerobic bacteria in the gut, may benefit the host physiology by limiting the overgrowth of potentially pathogenic bacteria such as *E. coli*, *Pseudomonas* and enterococci (Roberfroid et al., 2010 ▶). The results from the present study indicated a significant imbalance between protective lactobacilli and pathogenic bacteria in the gut. The significant shift towards overgrowth of pathogenic bacteria, especially *Pseudomonas* and *E. coli* suggests potential for alterations in gut physiology, such as the stimulation of inflammatory processes. In support of the present findings, Liu et al. (2014) ▶ had earlier shown that oral administration of Cd to mice resulted in significant inhibition of the growth of Lactobacillus and Bifidobacterium. Interestingly, treatment of Cd-exposed rats with NSO in the present study prevented the overgrowth of pathogenic staphylococci, *E. coli*, *Pseudomonas* and enterococci. This study shows, for the first time, that NSO could regulate microbiota composition in favor of anaerobic probiotic bacteria and thereby protect the gut.

The intestinal mucus, though often underestimated, is a key player in maintenance of gut homeostasis. The interaction of environmental pollutants with intestinal mucus and mucus-degrading microbial species, the so-called “mucophilic” bacteria, has become an interesting subject of efforts in assessment and management of environmental pollution (Gillois et al., 2018 ▶). The mucus layer of the gut, a complex secretion made up predominantly of water and glycoproteins, normally provides lubrication and protection of the underlying mucosa against mechanical damage from chemicals, toxins, carcinogens, pathological bacteria and reactive oxygen species (Phillipson et al., 2002 ▶). Crucially, the colonic mucus layer provides hospitable habitation for commensal bacteria, which bind to specific glycans and utilize mucins (high molecular weight glycoproteins produced by epithelial tissues), as an energy source (Kim and Khan, 2013 ▶). MUC-2 is the predominant mucin expressed in the small intestine and colon by goblet cells and its deficiency has been associated with the progression of colon disorders such as colon adenocarcinomas and spontaneous development of colitis (Hsu et al., 2017 ▶; Grondin et al., 2020 ▶).

The data presented in this article indicated reduction in goblet cell counts and PAS positivity in the colonic mucosa of rats treated with Cd alone, which were suggestive of reduced mucus production. This result was corroborated by decreased levels of colonic MUC2 protein in the Cd-treated rats. The exact mechanism by which this occurred was not elucidated in the present study and further studies are needed to clarify this phenomenon. It is, however, important to note that reduced mucus production in Cd-treated rats appeared to correlate with an increase in the growth of pathogenic bacteria, which contributed to the overall bacterial population in the gut. Among other factors, the gut microbiota composition is known to play major roles in influencing mucus properties as well as driving changes in the mucus layer. While both commensal and pathogenic bacteria are capable of degrading mucus, the activity of the latter, with depletion of the mucus layer, may lead to pathogen invasion and infection (Donaldson et al., 2018 ▶). Therefore, the plausible explanation for the Cd-induced reduction in colonic mucus content was the overgrowth of mucus-degrading bacteria, which utilized mucus glycans as main energy source (Paone and Cani, 2020 ▶). On the other hand, oral treatment with NSO was found to protect the colonic mucosa by stimulation of MUC2 expression and increased mucous production. Probiotics such as *Lactobacillus* spp have been shown to stimulate MUC2 production and secretion (Sicard et al., 2017 ▶), and this may explain the higher content of mucus production and higher expression of MUC2 in the colon of control and NSO-treated rats. It has also been found that short-chain fatty acids e.g. butyrate and acetate produced during bacterial fermentation in the gut, may stimulate MUC2 expression and thus, increase mucus secretion (Venegas et al., 2019 ▶). The involvements of transcription factors such as Nuclear factor kappa B (NF-κB) and mediators such as prostaglandins and nitric oxide in the regulation of secretion of the mucus layer have also been described (Schreiber et al., 2013 ▶), and it seems plausible that NSO could possibly act by stimulating components of the different pathways to increase mucus production and secretion.

Injury to tissues is normally accompanied by an inflammatory response with the secretion of several cytokines and the regulation of immune function. The tumor necrosis factor alpha (TNFα), a pro-inflammatory cytokine, is one of the earliest mediators in the inflammatory response and is often used as an independent index of inflammation during the development of tissue injury. Its secretion from activated macrophages is stimulated upon the entry of an inciting agent and the cytokine then activates other inflammatory cells, including neutrophils and lymphocytes, while also promoting the secretion of other cytokines (Parameswaran and Patil, 2010 ▶). Interleukin-2 (IL-2) is produced by helper T cells as an immune regulator, which among other functions, promotes the secretion of interferons, enhances secretion of antibodies from B cells and stimulates proliferation of T cells (Wang et al., 2016 ▶). Reduction in IL-2 levels usually indicates a suppression of the body’s cellular immune function (Jiang et al., 2017 ▶). Concerning the inflammatory status of the colon, the present findings indicated that Cd treatment increased colonic TNFα levels, while suppressing IL-2 expression. Conversely, NSO administration had the opposite effects, causing reduction in TNFα levels and increasing the levels of IL-2. Similar to our findings, dose-dependent reductions in serum IL-2 levels have been previously reported in male rats treated with Cd chloride (Elhady et al., 2012 ▶). It would, thus appear that the gut-protective activities of NSO against Cd-induced toxicity could involve both anti-inflammatory and immuno-stimulatory effects. The anti-inflammatory and immunomodulatory properties of *N. sativa* oil have been well documented and it is reasonable to suggest that these properties appeared to have encouraged a relatively stable level of microbial balance and mucin production in the colons of the rats (Ahmad et al., 2013 ▶). 

The main bioactive components identified in NSO by GC-MS analysis were unsaturated fatty acids, including trans-13-octadecenoic acid, E, Z-1,3,12-nonadecatriene-5, 14-diol and oleic acid, along with the well-known active ingredient of *N. sativa* seeds, thymoquinone and some terpenoids. According to Isik et al. (2019) ▶, GC-MS analysis of commercial black cumin seed oils, the major compounds identified were also fatty acids, including linoleic, oleic and palmitic acids. The important role of fatty acids in the modulation of intestinal barrier function is in gaining considerable attention, although there is as yet no consensus on the differential effects of saturated and unsaturated fatty acids on the intestinal barrier. However, based on accumulated evidence, what seems to be widely accepted is that the promotion of overall gut and microbiota health is encouraged by consumption of mono- and poly-unsaturated fatty acids, while saturated fatty acids should be avoided in the diet (Candido et al., 2017 ▶). Oleic acid, for example, has been reported to exert antioxidant and anti-inflammatory actions which were thought to mediate its gastro-protective effects in rats (Alzoghaibi, 2007 ▶). Short-chain fatty acids (SCFAs) such as butyrate and acetate have been reported to increase mucus secretion and MUC2 expression (Alzoghaibi, 2007 ▶). Very importantly, thymoquinone (2-isopropyl-5-methyl-1, 4-benzoquinone), the bioactive component of NSO is well known for its antioxidant, anti-inflammatory, and immunomodulatory activities (Chaieb et al., 2011 ▶; Dajani et al., 2016 ▶). It seems likely that the protective effects of NSO on the intestines, and especially the colon, were mediated by a combination of the activities of the major bioactive components including thymoquinone and the different fatty acids identified in the oil.

In summary, the present study provided profound insights into the mechanisms of intestinal protection by *N. sativa* oil against Cd-induced changes in microbiota, inflammatory status, mucus content and mucosal morphology. The mechanisms of NSO’s protection involved restoration of the balance of gut bacteria in favor of probiotic lactobacilli, increased expression of colonic MUC2, inhibition of pro-inflammatory TNFα and/or promotion of immunomodulatory effects of IL-2. This study suggests that *N. sativa* oil may be a useful therapeutic agent for the protection of metal (Cd)-induced intestinal toxicity.

## Conflicts of interest

The authors have declared that there is no conflict of interest.
